# Optical and Spin Properties of NV Center Ensembles in Diamond Nano-Pillars

**DOI:** 10.3390/nano12091516

**Published:** 2022-04-29

**Authors:** Kseniia Volkova, Julia Heupel, Sergei Trofimov, Fridtjof Betz, Rémi Colom, Rowan W. MacQueen, Sapida Akhundzada, Meike Reginka, Arno Ehresmann, Johann Peter Reithmaier, Sven Burger, Cyril Popov, Boris Naydenov

**Affiliations:** 1 Department Spins in Energy Conversion and Quantum Information Science (ASPIN), Helmholtz-Zentrum Berlin für Materialien und Energie GmbH, Hahn-Meitner-Platz 1, 14109 Berlin, Germany; kseniia.volkova@helmholtz-berlin.de (K.V.); sergei.trofimov@helmholtz-berlin.de (S.T.); rowan.macqueen@helmholtz-berlin.de (R.W.M.); 2 Institute of Nanostructure Technologies and Analytics (INA), Center for Interdisciplinary Nanostructure Science and Technology (CINSaT), University of Kassel, Heinrich-Plett-Str. 40, 34132 Kassel, Germany; j.heupel@ina.uni-kassel.de (J.H.); reithmaier@ina.uni-kassel.de (J.P.R.); popov@ina.uni-kassel.de (C.P.); 3 Zuse Institute Berlin, Takustraße 7, 14195 Berlin, Germany; betz@zib.de (F.B.); colom@zib.de (R.C.); burger@zib.de (S.B.); 4 Institute of Physics, Center for Interdisciplinary Nanostructure Science and Technology (CINSaT), University of Kassel, Heinrich-Plett-Str. 40, 34132 Kassel, Germany; s.akhundzada@physik.uni-kassel.de (S.A.); reginka@physik.uni-kassel.de (M.R.); ehresmann@physik.uni-kassel.de (A.E.); 5 Berlin Joint EPR Laboratory, Fachbereich Physik, Freie Universität Berlin, 14195 Berlin, Germany

**Keywords:** NV centers, diamond nano-pillars, fluorescence lifetime, ion implantation, optically detected magnetic resonance (ODMR), spin coherence time, spin relaxation time

## Abstract

Nitrogen-vacancy (NV) color centers in diamond are excellent quantum sensors possessing high sensitivity and nano-scale spatial resolution. Their integration in photonic structures is often desired, since it leads to an increased photon emission and also allows the realization of solid-state quantum technology architectures. Here, we report the fabrication of diamond nano-pillars with diameters up to 1000 nm by electron beam lithography and inductively coupled plasma reactive ion etching in nitrogen-rich diamonds (type Ib) with [100] and [111] crystal orientations. The NV centers were created by keV-He ion bombardment and subsequent annealing, and we estimate an average number of NVs per pillar to be 4300 ± 300 and 520 ± 120 for the [100] and [111] samples, respectively. Lifetime measurements of the NVs’ excited state showed two time constants with average values of τ_1_ ≈ 2 ns and τ_2_ ≈ 8 ns, which are shorter as compared to a single color center in a bulk crystal (τ ≈ 10 ns). This is probably due to a coupling between the NVs as well as due to interaction with bombardment-induced defects and substitutional nitrogen (P1 centers). Optically detected magnetic resonance measurements revealed a contrast of about 5% and average coherence and relaxation times of T_2_ [100] = 420 ± 40 ns, T_2_ [111] = 560 ± 50 ns, and T_1_ [100] = 162 ± 11 μs, T_1_ [111] = 174 ± 24 μs. These pillars could find an application for scanning probe magnetic field imaging.

## 1. Introduction

Diamond is a promising material for quantum information technology due to its unique physical properties. One of the main advantages is that it can host various optically active defects, which find applications as single-photon sources, quantum memories, and quantum sensors. The most prominent and best-studied color center in diamond is the nitrogen-vacancy (NV) center, as it represents a multi-purpose sensor for magnetic [1,2] and electric fields [3], temperature [4,5], and pressure [6], with high sensitivity and nano-scale spatial resolution. An amazing feature of the NV centers is that they can be optically observed at the single-site level and their fluorescence depends on the spin state of the NVs’ triplet ground state. This enables single-spin spectroscopy and magnetometry at ambient conditions. The main disadvantage of diamond as a host material is its high refractive index (2.42), which leads to a high total internal reflection at the air interface, thus limiting the number of detected photons emitted from the color centers. A possible solution to this problem is to embed the color centers in a photonic structure such as solid immersion lenses (SILs) [7], photonic crystals [8], and nano-pillars [9,10,11]. The last could also be used as tips for scanning probe magnetic field imaging [12]. Often, NV centers in nanodiamonds are used instead, but their crystal orientation is not well-defined and they also show a large size distribution. Usually, the goal is to introduce single NV centers in these structures, but often an ensemble of NVs is desired, which is also the focus of the current work. This can be applied for wide-field imaging applications, as reported recently [13].

There are three common pathways to fabricate NV center ensembles—creation during the diamond crystal growth, nitrogen ion implantation in diamond with low nitrogen concentration (type IIa), and ion/electron irradiation of nitrogen-rich diamond (type Ib, with typically 100 ppm P1 centers, i.e., electrically neutral single substitutional nitrogen atoms). For some applications, it is desired to use an ensemble of N NV centers. In this case, the magnetic field sensitivity increases as √N compared to a single NV and also all NV orientations are present, thus allowing determination of not only the strength, but also the direction of the magnetic field. The increased number of emitted photons could allow a simpler experimental setup, compared to the typically used confocal microscope, for example, a wide-field microscope [13]. Finally, using ensembles in pillars allows scanning probe magnetometry, though with limited spatial resolution compared to single NVs.

Here, we report on the fabrication of pillars with ensembles of NV centers in the two most common diamond crystal orientations—[100] and [111]. The purpose was to investigate how (and if) the pillar diameter would influence the spin and optical properties of the NVs. The goal was to find out which is the main process involved here—the fabrication of the pillars, the interaction among the NV centers, and/or the interaction with other defects. We used 6 keV He^+^ ion bombardment of type Ib samples for creating vacancies, which diffuse during the annealing process to form NV centers. The diamond nano-pillars were fabricated using electron beam lithography (EBL) and inductively coupled plasma reactive ion etching (ICP-RIE) of He-bombarded diamonds. The fabrication procedure and the applied parameters are well-established in our former works [14,15]. The NV center ensembles in the nano-pillars were characterized with respect to their optical and spin properties, which were compared to those of single NV centers in bulk diamond.

## 2. Materials and Methods

**Figure 1:**
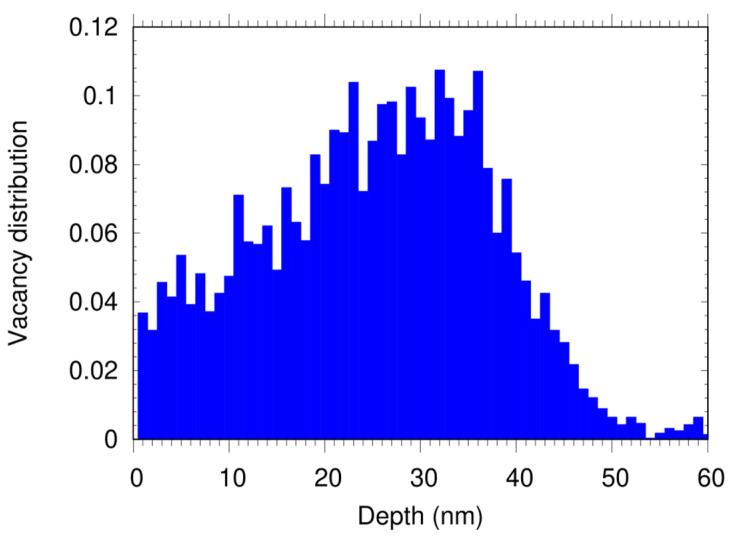
SRIM simulation for the creation of vacancies by 6 keV He^+^ ion bombardment under normal incidence. The NV centers created during the annealing process have the same depth profile.

We used type Ib diamond samples (Sumitomo) with [100] and [111] crystal orientations synthetized via the High-Pressure High-Temperature (HPHT) method [16]. The P1 concentration was measured using EPR spectroscopy and was found to be 68 and 65 ppm, respectively. The samples were bombarded by He ions with an energy E = 6 keV and a dose of 8 × 10^13^ ions/cm^2^ to create the vacancies. The depth-distribution of the latter, shown in Figure 1, was simulated with the SRIM software [17] using a displacement energy of the carbon atom of 37.5 eV [18]. This simulation treats the diamond as amorphous material, where the crystal structure is not considered. Such approximation is usually consistent with the experiments, but at low energies (below 10 keV) there are often discrepancies [19]. It has been reported that helium ion implantation in diamond creates about a four times higher number of vacancies in the [100] crystal orientation compared to [111] [20]. Here, we indirectly observed this effect, since the fluorescence measured from the [111] samples is lower compared to the [100] (see below), although the same ion dose was used. Since the nitrogen concentration in both samples is the same, we can conclude that the number of NVs in the [111] diamond is lower at least by a factor of 4, due to the lower number of created vacancies. 

**Figure 2:**
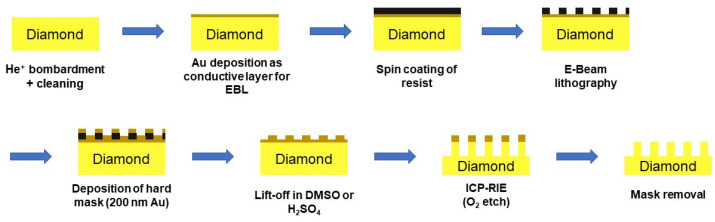
Scheme with the individual steps for the fabrication of nano-pillar arrays in single-crystalline diamond samples.

After the ion bombardment, the samples were annealed at t = 1000 °C in ultra-high vacuum (<10^−7^ mbar) for two hours in order to induce the migration of the vacancies and consequently the formation of NV centers. The implanted and annealed samples were cleaned with solvents (acetone, isopropyl alcohol (IPA), MicroChemicals GmbH, Ulm, Germany) and in a piranha acid solution (3:1 H_2_SO_4_:H_2_O_2_, 1 h, Technic France, Saint Denis, France) to remove any organic contaminations. The nano-pillars were defined by EBL (eLine, Raith GmbH, Dortmund, Germany). For this, a 7 nm Au layer has been deposited by electron beam evaporation (BAK 600, Oerlikon Balzers Coating, Bingen, Germany) as a conductive layer. Then, a positive EBL resist (AR-P 617.06, AllResist GmbH, Strausberg, Germany) was spin-coated on top of the diamond samples, followed by the definition of the pillar arrays. Due to the smaller size of the [111] sample (2 × 2 × 1.6 mm^3^) in comparison to the [100] sample (3 × 3 × 1.5 mm^3^), a higher rotational speed was chosen for this sample (3000 rpm for 30 s instead of 1500 rpm) and the resist was spin-coated twice to achieve sufficient thickness. The pillar design included circles with nominal diameters of 100 and 200 nm and a center-to-center distance of 10 µm. Different electron doses (42 μC/cm^2^–12.6 mC/cm^2^) were tested to minimize EBL proximity effects affecting the actual pillar diameter as well as to obtain pillars with various diameters. The proximity effect is the phenomenon of undesired exposure of areas adjacent to the scanned pattern due to the interactions of the primary electrons with the resist and the substrate. As a result of the electron forward scattering and backscattering, the resist outside the scanned pattern receives a non-zero dose, leading to enlargement of the structures, irrespective of the distance between them. The irradiated resist was developed with a mixture of methyl isobutyl ketone (MIBK, Technic France, Saint Denis, France) and IPA (1:3, 165 s) and then immersed in IPA for 30 s. As a hard mask for fabrication of the nano-pillars, a 200 nm gold layer (with 5 nm Ti as an adhesion layer) was deposited (BAK 600) on top of the structured resist. Subsequently, a lift-off process was performed either in dimethyl sulfoxide (DMSO, MicroChemicals GmbH, Ulm, Germany) at 80 °C overnight or with sulfuric acid (96% H_2_SO_4_, 5 min), so that only the round pillar features remained on the sample surface. To transfer these features into the diamond sample, an ICP-RIE system (PlasmaLab 100, Oxford Instruments plc, Abingdon, United Kingdom) was utilized. Oxygen was applied as reactive gas, and the parameters for the dry etching procedure were 1000 W ICP power, 200 W RF power, 10 sccm O_2_ flow, 30 °C substrate temperature, and 5 mTorr (0.7 Pa) chamber pressure. To fabricate 1 to 2 µm-high pillars with a small diameter at the apex, the etch time was set to 11 min for the [100] sample and 8 min for the [111] one. After the etching process, the Au mask was removed with potassium iodide solution (4:1 mixture of KI:I_2_) and the Ti adhesion layer with hydrofluoric acid solution (10% HF). Regarding the morphology, the quality of the structures, and the size examinations, the pillar arrays were investigated with a scanning electron microscope (SEM,S-4000, Hitachi, Krefeld, Germany). With the software ImageJ (Ver. 1.8.0), the pillar dimensions (height and apex diameter) were determined by measuring pixel distances in the distinct SEM images. A systematic error of 10% was estimated for each value. The entire process for fabrication of diamond nano-pillars is sketched in Figure 2.

Finite element analysis was performed using the JCMsuite software. The optical characterization of the NV centers was carried out on a custom-made confocal microscope having continuous wave (CW) and pulsed microwave excitation for performing optically detected magnetic resonance (ODMR) experiments. For confocal scanning and ODMR, we used a diode laser (iBEAM-SMART-515-S, TOPTICA Photonics AG, Munich, Germany) with a wavelength of 515 nm, with an air objective (Olympus UPLAN FL 100×−0.95, Olympus Deutschland GmbH, Hamburg, Germany) with NA = 0.95. The laser light was blocked from entering the detector by a 650 longpass filter (Thorlabs FELH0650, Thorlabs GmbH, Bergkirchen, Germany). The fluorescence spectra were measured using an IsoPlane 160 Imaging Spectrograph equipped with a Princeton Instruments PIXIS:100B eXcelon Liquid Cooled Digital CCD. For these experiments, we used a 550 longpass filter (Thorlabs FELH0550, , Thorlabs GmbH, Bergkirchen, Germany). The laser source for the lifetime measurements was an NKT SuperK Fianium supercontinuum laser (λ = 550 nm) with an adjustable repetition rate and user-selectable output wavelength band (Varia, NKT Photonics GmbH, Cologne, Germany). For time-correlated photon-counting measurements (life time), we used an avalanche photo diode (Excelitas SPCM-AQRH-14, Qioptiq Photonics GmbH & Co. KG, Wiesbaden, Germany) and a TimeHarp 260 Nano (PicoQuant, Berlin, Germany) as a counter. For comparing the count rates with single NVs, neutral density filters were used when measuring the pillars, otherwise the APD is saturated. For controlling the experiments and data acquisition, we used the Qudi software suite [21]. 

## 3. Results

**Figure 3:**
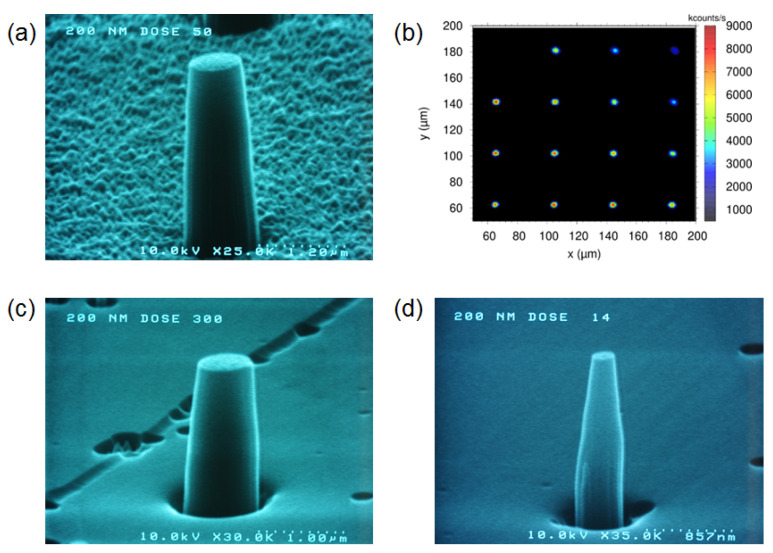
(**a**) SEM micrograph of a single pillar in the [100] diamond sample (2.1 mC/cm^2^, diameter approx. 720 nm). (**b**) Confocal microscopy image of the diamond nano-pillars, showing the fluorescence of NV centers. SEM micrographs of pillars in the [111] sample, written with different electron doses: (**c**) 12.6 mC/cm^2^, diameter approx. 600 nm, in pillar design round feature of 200 nm; (**d**) 588 µC/cm^2^, diameter approx. 210 nm, in pillar design round feature of 200 nm.

An SEM micrograph of a typical diamond nano-pillar in the [100] sample is depicted in Figure 3a. With high electron doses in the range of 1.3 mC/cm^2^–12.6 mC/cm^2^, pillars with diameters between 670 and 980 nm, i.e., larger than the nominal ones, were obtained. A fluorescence map from a nano-pillar array is shown in the confocal microscope image of Figure 3b. The fluorescence spots resemble the pattern of the pillar array designed by the fabrication process. In some cases, single dots in the fluorescence pattern are missing, which could be due to removal either of the shallow layer with NV centers (at a depth up to 50 nm, as revealed by the SRIM calculation, see Figure 1) during the etching process, or of the hard mask spots after the lift-off procedure. In comparison to the nano-pillars fabricated on a [111] sample (Figure 3c,d), the area between the pillars in the [100] sample appears rougher. This is due to residuals of the Au conductive layer after the lift-off process. Presumably, the nanometer-thin gold film wrinkled on top of the diamond surface during the bake-out of the electron sensitive resist at 250 °C. By etching, this wrinkled pattern was transferred into the diamond, leading to the observed rough grainy appearance of the surface. For the [111] sample, the conductive Au layer was completely removed during the lift-off process, resulting in a smooth surface between the pillars after the etch step. Noticeable is the low density of etch pits, in particular, triangular-shaped ones located at step defects from the original surface, which are typical defects after etching for diamond samples with this orientation [22,23].

In the case of the [111] sample, the conductive Au layer as well as the application of a thicker electron resist (by repeated spin coating) were beneficial for the alignment in the EBL process, leading to a more precise focusing of the electron beam without charging effects. Therefore, the diameters of the pillars written with the same dose are smaller for the [111] sample, e.g., with a max. dose of 12.6 mC/cm^2^ the pillar diameter is ~610 nm for the [111] and about 980 nm for the [100] sample. With smaller doses and hence with smaller lithography features, the actual diameter approaches the nominal one, however, the nano-pillars appear more tapered, which can be explained by the more conical shape of the Au mask before the etching procedure. The initial mask shape is then transferred into the diamond nano-pillar structure, since the slope angle of the hard mask is amplified and the mask is continuously removed by erosion [24]. Due to this effect (so-called differential dry etching), nano-pillars with apexes down to 15 nm (electron dose 420 µC/cm^2^ and 100 nm nominal diameter in pillar design) can be fabricated. Additionally, the heights of the fabricated pillars were approx. 2.2 µm ± 0.2 µm for the longer etched [100] sample and approx. 1.4 ± 0.2 µm for the shorter etched [111] sample, as measured from the SEM images, from which etching rates of 180–220 nm/min and 150–200 nm/min, respectively, were determined.

**Figure 4:**
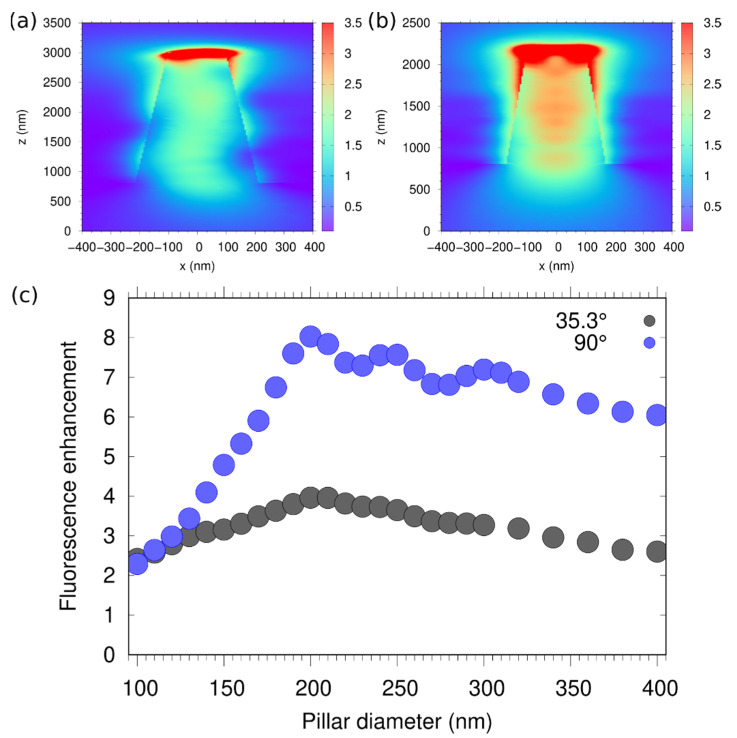
Electric field distribution of incoherently superimposed dipoles oriented in an angle θ of 35.3° (**a**) and 90° (**b**) with respect to the surface normal. (**c**) Enhancement of the emitted fluorescence into the objective (NA = 0.95) as a function of the pillar diameter compared to the corresponding collection of dipoles below a flat interface (θ = 35.3°).

We use the finite-element method to quantify the effect of the pillars on the fluorescence into the objective (NA = 0.95, see Figure 4). Incoherently superimposed dipole sources, uniformly distributed 30 nm below the upper surface, give rise to electric field distributions (Figure 4a,b) that depend on the geometry of the pillar and the angle θ between dipole moment and surface normal. In the simulations, the emission wavelength was set to λ = 700 nm. The alignment of the NV center limits the number of possible dipole orientations. We select two typical angles (35.3° and 90°) and evaluate the enhancement of the fluorescence into the objective as a function of the pillar diameter (taken at the top of the pillar). The references are equally sized collections of dipoles below a flat surface (θ = 35.3°). Figure 4c shows a significant enhancement of the fluorescence into the objective for both dipole orientations, with its maximum at a diameter of about 200 nm. For comparison, the plane interface combined with the ideal angle of 90° results in an enhancement factor of 2.5, which both orientations in the pillar exceed for a wide range of diameters. Further simulations (data not shown) allow the enhancement to be attributed to an improved collection efficiency. This is in agreement with previously reported results for similar nano-structures [13].

**Figure 5:**
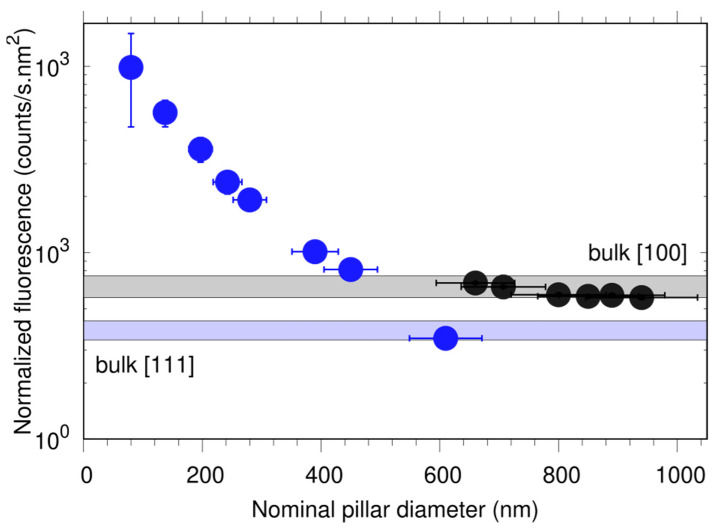
Fluorescence signal from the pillars as a function of the nominal pillar diameter in the [111] (blue) and [100] (black) samples. The measured photon count rate was kept below 10 MCounts/s using a neutral density filter in order not to saturate the APD. The light blue and grey rectangles show the estimated number of NVs in the bulk structures. See text for details.

The fluorescence signal as a function of the nominal diamond pillar diameter for both samples is shown in Figure 5. In order to compare the influence of the different pillars on the fluorescence emission, we normalize the latter over an area of 1 nm^2^. Here, we assume that the NV distribution over the sample surface is uniform.

We observe that the normalized fluorescence decreases with increasing diameter and it becomes constant for values above 600 nm. A possible explanation could be that the size of the laser spot (approximately 660 nm) becomes comparable with the diameter of the pillars and thus not all emitted photons from the NV centers in pillars with larger diameters can be collected. This conjecture is supported by measuring the fluorescence from larger structures (bulk areas, few tens of microns, used as markers for identifying the pillar arrays) on both samples (see also Figure 5). The results are in good agreement with the theoretical simulations (see Figure 4c), suggesting that indeed there is enhanced photon collection efficiency due to the pillar nanostructure. There is a discrepancy between the simulations and measurement data for pillars with diameters below 200 nm, where we observe much higher fluorescence signal than suggested by the calculations. A possible explanation would be limitation in our theoretical model (e.g., single-wavelength emission, two dipole orientations) and/or error in estimating the pillar diameter. However, this enhancement is not caused by a Purcell effect, since the latter is not expected from our simulations (data not shown) and also not observed in the lifetime measurements, as seen below. 

We estimate the number of NV centers in the pillars by comparing their fluorescence signal with the count rates from a single NV center (using the same laser power) in another diamond sample (type IIa, from Element Six). The single NV is fabricated by low-energy nitrogen ion implantation (E = 2 keV) and it is close to the diamond surface, which allows to compare the photon emission. For the [111] sample, we assume that the number of created NV centers is lower by a factor of 4, due to the lower number of vacancies created during the ion bombardment (see above). This procedure would not give the precise number of NV centers (see for example [11]), but it still provides a good estimation. The average number of NVs per pillar is estimated to be 4300 ± 300 and 520 ± 120 for the [100] and [111] samples.

**Figure 6:**
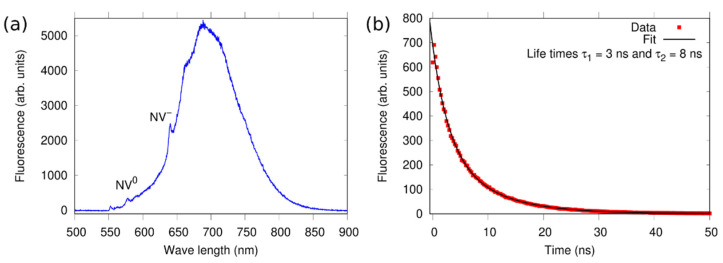
(**a**) Typical fluorescence spectrum from an exemplarily chosen single pillar, where the zero-phonon line of NV^-^ centers at 637 nm is clearly visible. The line at 575 nm suggests the presence of a small number of NV^0^ centers. (**b**) Lifetime measurement of the NVs’ excited state in a pillar, revealing a bi-exponential decay with time constants τ_1_ = 3 ns and τ_2_ = 8 ns. The bulk areas of both samples show similar spectra and lifetime values (data not shown).

The fluorescence spectra of all measured pillars showed the typical line shape with a maximum intensity at around 700 nm and a zero-phonon line (ZPL) of the NV^-^ centers at λ = 637 nm. A typical spectrum is plotted in Figure 6a.

Fluorescence lifetime measurements of all investigated pillars are best fitted with a bi-exponential decay function, with average decay constants τ_1_ [100] = 2.3 ± 0.4 ns and τ_2_ [100] = 8.1 ± 0.7 ns, and τ_1_ [111] = 2.5 ± 0.2 ns and τ_2_ [111] = 9.5 ± 1.3 ns for the [100] and [111], respectively. These are shorter compared to that of single NV centers, where we observe a mono-exponential decay with average τ = 9.5 ± 1.1 ns. A typical data set is shown in Figure 6b. The shorter lifetime values, however, are not a signature of Purcell effect, meaning that we do not observe enhanced photon emission from the pillars. This is confirmed by theoretical simulations (data not shown) as well as by lifetime measurements on the larger structures on both samples, where we find τ_1_ [100] ^bulk^ = 2.3 ± 0.3 ns and τ_2_ [100] ^bulk^ = 7.6 ± 0.6 ns, and τ_1_ [111] ^bulk^ = 1.9 ± 0.2 ns and τ_2_ [111] ^bulk^ = 6.5 ± 0.4 ns. Similar values have been previously reported in Ib diamonds, where the NV centers were created using a helium ion microscope [25] or via neutron irradiation [26]. In these studies, the authors suggested that the presence of other defects, created during the irradiation, lead to the observed shortened lifetime and to the bi-exponential decay.

**Figure 7:**
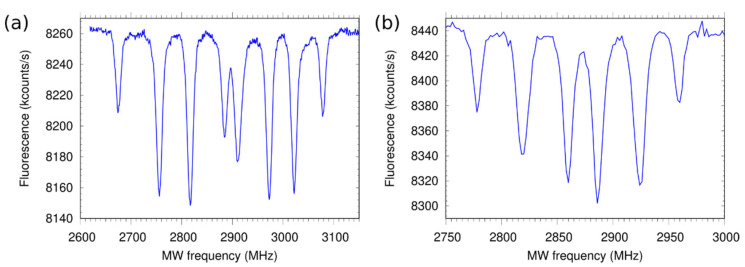
ODMR spectra of the NV centers in the (**a**) [100] and (**b**) [111] samples. The applied magnetic field is B_0_ [100] = 36.5 G and B_0_ [111] = 32.5 G, respectively. The different contrast of the transitions is probably due to varying microwave field for different crystal orientations of the NVs. The bulk structures show similar ODMR spectra (data not shown).

In Figure 7, ODMR spectra of NV ensembles in both samples are plotted. The eight spectral lines arise from the four possible crystal orientations of the NVs (in the case of [111] two of them overlap), showing that there is no preferential orientation of NV centers, as expected for bombardment-induced NV creation. The ODMR contrast varies probably due to different microwave field magnitudes for the four crystal orientations of the NV centers. The maximum ODMR contrast is about 5%. 

We performed pulsed ODMR measurement (data not shown) inversion recovery and Hahn echo decay to determine the electron spin relaxation (T_1_) and coherence (T_2_) times of the NV spins, and found them to be T_1_ [100] = 162 ± 11 μs, T_1_ [111] = 174 ± 24 μs and T_2_ [100] = 420 ± 40 ns, T_2_ [111] = 560 ± 50 ns. Similar values are observed for the bulk structures. While the T_2_ values are typical for NV centers in nitrogen-rich diamond crystals, T_1_ is much shorter than the expected value of several milliseconds. This result could be explained by the presence of some fast (GHz time scale) fluctuating paramagnetic defects in the vicinity of the NV centers and could be also due to interaction among the NVs electron spins themselves. The coherence time can be significantly prolonged (at least by an order of magnitude) if the NV centers are created via nitrogen doping during the chemical vapor deposition (CVD) growth of the diamond crystal, as demonstrated recently [27].

## 4. Discussion

The fabricated NV centers in the diamond nano-pillars show a typical fluorescence spectrum for negatively charged NVs, though there is a small contribution from NV^0^. The fluorescence lifetime decays are best fitted with a bi-exponential function, where the values are shorter compared to that of a single NV center. Since we can exclude the Purcell effect (see above), the shortened life time is probably due to the presence of defects created by the helium ion bombardment. Electron-irradiated (also helium-bombarded) nanodiamonds, which usually consist of a similar diamond material, often show longer lifetimes (compared to NVs in bulk crystals) when their size becomes much smaller (<100 nm) than the wavelength of the emitted light. This can be explained by the reduced number of electromagnetic field modes, leading to a lower coupling to the excited state, resulting in fewer decay channels for relaxation from the excited to the ground state.

The ODMR spectra show eight resonance lines (in the spectrum of the [111] sample, some of them overlap), corresponding to the four different crystal orientations of the NV centers as expected. Using an ensemble of NVs allows measurement of not only the strength of the magnetic field, but also its direction [28]. The large number of NV centers in a single pillar results in a stronger fluorescence, which speeds up the detection and improves the sensitivity [29]. We measure a photon count rate up to 10^7^ counts/s (by removing the ND filter, values up to 2.5 × 10^8^ counts/s are expected) from a single pillar without reaching saturation due to the limited power of our laser. By considering the average number of NVs in the pillars and their electron spin coherence time of 0.4 and 0.56 μs, we calculated (according to [29]) maximum achievable magnetic field sensitivities of $n_{AC}^{\left[100\right]}\approx 11.6\;nT/\surd Hz$ and $n_{AC}^{\left[111\right]}\approx 26.8\;nT/\surd Hz$ for the [100] and [111] samples, respectively.

## 5. Conclusions

Our method for nano-fabrication of diamond pillars with an ensemble of NV centers relies on HPHT samples with a high concentration (about 100 ppm) of substitutional nitrogen, also known as P1 centers. This material is widely used in the industry for grinding and cutting processes, and it is readily available and much cheaper compared to other type of diamond crystals. Therefore, the results presented here would allow a low-cost production of nano-structured diamond quantum sensors for various applications. The expected magnetic field sensitivity of our nano-pillars is much lower, compared to the state-of-the-art NV centers in diamond, but it would be still useful for various wide-field and scanning probe applications, where relatively strong fields have to be measured, for example, when magnetic layers or hard drives are investigated.

## Data Availability

The data from this study is available upon request.
